# Ribosomes: An Exciting Avenue in Stem Cell Research

**DOI:** 10.1155/2020/8863539

**Published:** 2020-07-06

**Authors:** Zhenzhen Han, Qi Zhang, Yanbo Zhu, Jingcheng Chen, Wei Li

**Affiliations:** Stem Cell and Cancer Center, The First Hospital of Jilin University, 71 Xinmin Street, Changchun, Jilin 130021, China

## Abstract

Stem cell research has focused on genomic studies. However, recent evidence has indicated the involvement of epigenetic regulation in determining the fate of stem cells. Ribosomes play a crucial role in epigenetic regulation, and thus, we focused on the role of ribosomes in stem cells. Majority of living organisms possess ribosomes that are involved in the translation of mRNA into proteins and promote cellular proliferation and differentiation. Ribosomes are stable molecular machines that play a role with changes in the levels of RNA during translation. Recent research suggests that specific ribosomes actively regulate gene expression in multiple cell types, such as stem cells. Stem cells have the potential for self-renewal and differentiation into multiple lineages and, thus, require high efficiency of translation. Ribosomes induce cellular transdifferentiation and reprogramming, and disrupted ribosome synthesis affects translation efficiency, thereby hindering stem cell function leading to cell death and differentiation. Stem cell function is regulated by ribosome-mediated control of stem cell-specific gene expression. In this review, we have presented a detailed discourse on the characteristics of ribosomes in stem cells. Understanding ribosome biology in stem cells will provide insights into the regulation of stem cell function and cellular reprogramming.

## 1. Introduction

Ribosomes are subcellular cytoplasmic biomolecules composed of rRNA and dozens of proteins. Ribosome sedimentation coefficients in eukaryotic cells and prokaryotic cells are 80S and 70S, respectively. Ribosomes primarily participate in translation, but recent research shows their involvement in multiple biological processes, such as cellular proliferation, differentiation, homeostasis, and development of cancer (these are known as “heterogeneous ribosomes”) [1, 2]. The ribosome filter hypothesis posits that, besides constituting the translation machinery, ribosomes influence the selective expression of mRNAs, thereby differentially regulating cellular function [3]. The efficiency of ribosome biosynthesis depends on specific environments, thereby differentially regulating the function of various cells, such as stem cells. Self-renewal is an attribute of stem cells that requires high translation efficiency [4–8]. Inhibiting translation of genes using transcriptional repressors leads to reduced stemness [4]. Hematopoietic stem cells also require significant ribosomal activity [9]. Cells can internalize ribosomes via trypsin-activated endocytosis to generate cell clusters similar to embryonic bodies expressing pluripotency markers [10]. It has been reported that ribosomes regulate stem cell differentiation and embryonic growth [11]; however, the mechanisms involved in this process remain to be understood. This review summarizes characteristics of “stem ribosomes”.

### 1.1. Ribosome-Mediated mRNA Translation

mRNA translation primarily involves 3 steps: initiation, elongation, and termination [12]. And the mRNAs have dynamic interactions of the small and large subunits of the ribosome, aided by multiple auxiliary factors during the process of translation [13]. Ribosomes read the codons (genetic code) in the mRNA; each codon corresponds to the addition of an amino acid [14]. Initiation is an important rate-limiting step in translation [15]. During this step, initiation factors facilitate the recruitment of the 40S subunit to the mRNA 5′ end, scanning of the 5′ untranslated region (UTR), start codon recognition and 80S subunit joining to form an elongation-competent ribosome [16–18]. mRNAs possess regulatory elements that regulate the frequency of translation initiation, choice of the open reading frame (ORF), global and local rates of elongation, and protein folding [19]. Structured or excessively short 5′ UTRs [20, 21] and upstream open reading frames (uORFs) [20, 22] negatively influence translation efficiency, while internal ribosome entry sites (IRESs) [23, 24], other regions of direct ribosomal recruitment [25, 26], and codon bias at the sites of initiation sites [27, 28] enhance initiation in response to ribosome shortage. The efficiency of elongation depends on codon usage, secondary structures in the mRNA, and ribosome density. Finally, translation terminates when the ribosome encounters a termination codon [19]. Thus, the cis-elements in mRNAs can be used in combinations to regulate the activity of ribosomes, thereby resulting in selective gene expression. This gives rise to ribosome heterogeneity that includes subsets of ribosomes with differential selectivity for mRNA subpools [2].

### 1.2. Assembly of Ribosomes

**Figure 1:**
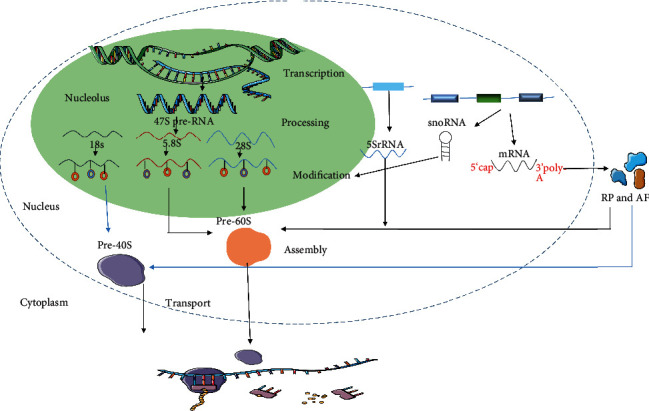
Eukaryotic ribosome synthesis. Eukaryotic ribosome synthesis is a complex process that comprises 5 steps, including transcription, processing, modification, assembly, and transport. (1) Transcription: RNA polymerase I transcribes rDNA into 47S preRNA. RNA polymerase III transcribes 5S rRNA. snoRNAs are transcribed by RNA polymerase II or III from non-protein-encoding regions or mRNA introns. RNA polymerase II transcribes the mRNAs for ribosome proteins (RPs) and assembly factors (AFs). (2) Processing: 47S pre-rRNA is processed to 18S, 28S, and 5.8S rRNAs. (3) Modification: there are two primary kinds of modifications on rRNA that are mediated by snoRNAs: 2′-O-methylation (2′-O-Me) and pseudouridines (*Ψ*). (4) Assembly: RPs and AFs are translated in the cytoplasm and shuttled to the nueclus for ribosome assembly. The pre-60S subunits comprise 28S, 5.8S, and 5S rRNA, and the pre-40S subunit includes an additional 18S rRNA. (5) Transport: the subunits are transported to the cytoplasm via the nuclear pore to be assembled as needed during translation.

Ribosome synthesis is an energy-intensive process that requires complex machinery comprising numerous proteins and RNAs (Figure 1) [29]. Ribosomes are assembled from large and small subunits: large and small subunits predominantly function in peptide bond transfer and mRNA decoding, respectively [30]. There are four main components of ribosome synthesis: ribosome proteins (RPs), assembly factors (AFs), ribosomal RNAs (rRNAs), and small nucleolar RNAs (snoRNAs) [1]. Ribosome precursors are synthesized in nucleoli whose internal structure comprises three characteristic regions: fiber center (FC), dense fiber component (DFC), and particle component. rRNAs are transcribed between FC and DFC. rRNAs and their binding proteins reside in the DFC. rRNAs are also cleaved, processed, and modified in the DFC. The ribosome precursor is assembled in the particle component [31]. In eukaryotic nucleoli, RNA polymerase I transcribes rDNA into 47S preRNA that is spliced to form 5.8S, 28S, and 18S rRNA [32, 33]. In the eukaryotic nucleus, RNA polymerase III transcribes 5S rRNA that participates in the formation of the 60S subunit with 28S and 5.8S rRNA. The 40S subunit is composed of 18S rRNA and 33 RPs, while the 60S subunit comprises 5S, 5.8S, and 28S rRNA and 47 RPs.

rRNAs can be modified or processed by snoRNAs [34] that are transcribed by RNA polymerase II or III or arise from pre-mRNA introns. snoRNAs are found in the nucleus and provide a direct role in the post transcription of rRNA and mRNA [35]. snoRNAs interact with proteins to form small nuclear ribonucleoproteins (snoRNPs) that direct rRNA processing and modification [36].

There are ~80 RPs [37], majority of which are cotranscribed with rRNA [38]. mRNAs for RPs are translated in the cytoplasm following which they are transported back to the nucleus to form the precursor of ribosomal subunits. To enable efficient protein translation, ribosome assembly also requires specific AFs [39]. Eukaryotes possess more than 500 AFs [40]. AFs are associated with rRNA at specific stages including rRNA processing and modification, thereby facilitating the binding of RP and influencing ribosome biogenesis [41]. AFs mainly consist of multiple enzymes and proteins with known protein or RNA-binding domains. Specific AFs such as FBL and BYSL are overexpressed in stem cells and maintain pluripotency by promoting ribosome biogenesis [42–44].

Differentiation of embryonic stem (ES) cells can be caused by a decrease in ribosomal abundance. Inhibition of protein synthesis influences numerous proteins with short half-lives. The expression of key proteins with short half-lives depends on multiple factors [45]. In human ES cells, the expression of the short-lived Nanog protein is erratic. The proteolysis of Nanog is mediated by the ubiquitin–proteasomal pathway [46]. Mouse embryonic stem cell (mESCs) can be treated with the transcription inhibitor 4EGI-1 to result in the rapid reduction of the protein levels of Nanog, Esrrb, and Tfcp2l1 and a steady time-dependent reduction in their mRNA levels [47]. Ribosome biogenesis is composed of five main steps involving transcription, processing, modification, assembly, and transport of ribosome precursors. Careful regulation of the multiple steps in ribosome biogenesis enables efficient translation and is critical for maintaining pluripotency.

### 1.3. Ribosome-Induced Cellular Transdifferentiation

Transdifferentiation involves the reprogramming of somatic cells into those of a different lineage without going through the intermediate proliferative pluripotent stem cell stage; it is a new method to generate functional cells [48–50]. *Mycobacterium leprae* transdifferentiates Schwann cells into pluripotent cells by downregulating differentiation markers (SOX10, Mpz, and p75) and upregulating genes associated with mesodermal development (Sox2, CD44, and CD43) [51]. *Helicobacter pylori* infection in intestinal epithelial cells promotes the expression of CDX1 [52]. CDX1 induces the expression of pluripotency factors KLF5 and SALL4, thereby transdifferentiating gastric epithelial cells into intestinal epithelial-like cell [52, 53]. Proteins from *Wolbachia pipientis*, especially W20, accelerate mammalian cell reprogramming [54]. Lactic acid bacteria (LAB) convert human dermal fibroblasts (HDFs) into pluripotent cells [55]. LAB-differentiated cell clusters have the potential to form three germ layer cells along with increasing the expression of the marker for pluripotency, Nanog [55]. Thus, bacteria promote host cell reprogramming, but the mechanisms involved remain to be investigated. To understand LAB-induced transdifferentiation of HDFs, LAB lysates were used to treat trypsinized HDFs; the protein fraction of size > 100 kDa obtained from ultrafiltered lysates was found to induce cell cluster formation [10]. Owing to the size of the fraction, the “transdifferentiation factor” was speculated to be the ribosome. Purified ribosomes obtained by ultracentrifugation promoted HDF transdifferentiation. These ribosome-induced cell clusters can enhance the expression of pluripotency factors and give rise to endodermal, mesodermal, and ectodermal cells, but they could not form teratomas and chimeras [10]. Ribosome-induced cell clusters need to be induced with trypsin [56]. Since the diameter of a ribosome is ~20 nm [57], it can undergo nucleocytoplasmic shuttling and internalized by other cells via endocytosis by endosomal vesicles that are ~10 *μ*m in size [58]. The characteristics of this ribosome that promote transdifferentiation and express stem cell markers remain to be understood fully.

### 1.4. rRNA Transcription Efficiency Determines the Fate of Stem Cells

The nucleus of ES cells quickly adapts to increases in cellular proliferation that requires rapid transcription of rRNAs [59, 60]. To promote the initiation of transcription, RNA polymerase I specifically binds to the promoter region of rDNA via transcription factors, such as upstream binding factor (UBF) and promoter selectivity factor (SL1/TIF-IB) [61]. The efficiency of rRNA transcription determines the speed of ribosome biosynthesis and assembly. Stem cells heavily transcribe rRNAs, but their levels decrease as cells differentiate [32]. The expression of c-Myc, an important stem cell marker, decreases during differentiation [62]. A reduction in the levels of RNA polymerase-associated factors downregulates rRNA synthesis [63], thereby inducing cell differentiation [64]. Downregulation of rRNA correlates with an increase in the levels of linage-specific factors that are responsible for differentiation into specific cell types (e.g., MyoD and myogenin during myogenesis, Runx2 during osteogenesis, and C/EBP-*β*, C/EBP-*δ*, and C/EBP-*α* during adipogenesis); these factors hinder rRNA transcription by interacting with UBF or rDNA promoters [65]. An ex vivo experiment demonstrated that actinomycin D-mediated inhibition of rRNA transcription induces the differentiation of mouse hematopoietic stem cells (HSCs). Thus, it is generally accepted that a decrease in rRNA transcription correlates with cellular differentiation.

In eukaryotes, 75% of rRNAs are transcribed by RNA polymerase I [1]. This enzyme complex comprises Udd, TAF1B, and a TAF1C-like factor in *Drosophila*. Increased transcription by RNA polymerase I inhibits cell differentiation, while inhibition of RNA polymerase I-mediated transcription limits ribosome biogenesis and promotes cellular differentiation [66]. FBL methylates a glutamine residue in histone H2A and stimulates RNA polymerase I binding on rDNA gene promoters [67].

A recent study has shown that 17 pluripotency-associated factors bind rDNA loci in mESCs [32]. Moreover, silencing of rDNA genes and downregulated ribosome biogenesis are associated with stem cell ageing in murine HSCs [68]. In general, stem cells have higher rRNA transcription efficiency than the daughter cells and rRNA synthesis is downregulated by phenotype-specific transcription factors during differentiation. rDNA transcription is quantitatively regulated in stem cells and the rate of rDNA transcription influences cell fate. Beyond rDNA transcription: many factors at all steps of the process appear to play stem cell-specific roles.

### 1.5. rRNA Processing and Stem Cells

rRNA processing is an evolutionarily conserved phenomenon that is essential for ribosome assembly. Ribosome assembly and pre-rRNA processing are closely linked, and the primary 47S transcript is cleaved to the 20S and 32S intermediates that are processed to the mature 18S and 5.8/28S rRNAs (components of the 40S and 60S ribosomal subunits, respectively). Stem cell AFs promote rRNA processing to improve the efficiency of ribosome synthesis. Small subunit processome (SSUP) is a pre-18S processing complex composed of snoRNA U3 and 54 proteins encoded by six genes (Krr1, Ddx47, Ddx52, Nol6, Pdcd11, and Rrp7a) in mESCs [4]. These SSUP genes are overexpressed in stem cells but downregulated during embryoid body formation. Depleting cells of the SSUP reduces Nanog expression, while knocking out SSUP genes hinder cellular reprogramming. Krr1, a conserved yeast homolog of SSUP [45], promotes the cleavage of 18S rRNA at sites A0, A1, and A2 to generate the 40S subunit [69]. SSUP stimulates pluripotency by enhancing translation. ES cells exhibit an upregulation in the subunits of SSUP, thereby enhancing the rate of translation and regulating pluripotency.

*Lrrc34* (leucine-rich repeat-containing 34) is another gene that is robustly expressed in mESCs and is downregulated during differentiation [70]. Lrrc34 is a nucleolar protein that interacts with nucleophosmin and nucleolin regulate pluripotency-related genes, such as OCT4, and is important in rRNA processing and ribosome formation [71]. Urb2, another nucleolar protein, plays a role in 27S pre-RNA processing and 60S subunit biogenesis [72]. Moreover, mutations in Urb2 impair HSC development by disrupting the biogenesis of ribosomal subunits and rRNAs in zebrafish [11, 72].

Nucleostemin is overexpressed in proliferating cells, such as central nervous system stem cells, ES cells, and cancer cell lines, and downregulated during differentiation. It contains an N-terminal basic domain that is involved in nucleolar localization and two GTP-binding motifs that regulate its transport between the nucleolus and nucleoplasm [73, 74]. Nucleostemin regulates cell proliferation via p53 signaling and is involved in ribosomal biogenesis, especially pre-RNA processing. It is a large protein complex (>700 kDa) comprising five ribosomal subunits (RPS6, RPS8, RPS24, RPL13, and RPL14), three nucleolar proteins (DDX21, Pes1, and EBP2), and a translation initiation factor (eIF2B1) [75]. DDX21 is a DExD/H box protein that uses energy from ATP hydrolysis to unwind RNA or disrupt RNA-protein complexes that could alter RNA [76]. It stabilizes 28S rRNA, promotes the conversion of the 20S pre-RNA into 18S RNA in *Xenopus*, and processes of 18S and 28S rRNAs in humans [77]. Pes1 is also involved in processing the 12, 36, and 32S pre-rRNAs in mammals, thereby promoting the biogenesis of the 60S ribosomal subunit [78]. EBP2 interacts with ribosomal proteins L36, L34, and L8; L36 is important for processing 27SA2, 27SA3, and 27SBL pre-rRNAs [79]. Furthermore, nucleostemin and 60S subunits can be found in the same fraction following sucrose gradient centrifugation, indicating the involvement of nucleostemin in ribosome synthesis [75]. In summary, the interactions between DDX21, Pes1, EBP2, and nucleostemin enhance pre-RNA processing to promote 60S ribosomal subunit synthesis and improve the efficiency of translation.

Bystin-like (BYSL) is detected in abundance in rapidly proliferating embryo and cancer cells and is evolutionarily conserved across eukaryotes, especially the C-terminus that regulates its nuclear localization [80–83]. Knocking out BYSL inhibits the synthesis of 18S rRNA and enables the accumulation of 20S rRNA precursors without affecting 28S rRNA. Moreover, there is a decrease in the cytoplasmic content of the 40S subunit, suggesting the role of Bysl in the export of the 40S subunit [33]. Bysl is also a key regulator of c-Myc and is overexpressed in stem and cancer cells [84, 85]. Enp1 is the yeast ortholog of Bysl that is predominantly localized to the nucleolus. Similar to Krr1, Enp1 functions in 18S rRNA processing and cleavage of the 35S pre-RNA at sites A0, A1, and A2 [86]. Enp1 has been observed to coimmunoprecipitate with a cohort of proteins, including Nop1 (the yeast ortholog of FBL) [87]. Enp1 and Nop1 interact with snoRNAs U3 and U14 and stimulate rRNA processing.

### 1.6. Specific rRNA Modifications in Stem Cells

rRNA modifications change according to different stimuli, diseases, and development, and this results in ribosome heterogeneity, thereby differentially regulating gene expression [34]. Eukaryotic rRNAs possess 91 pseudouridines (*Ψ*), 105 sugars containing 2′-O-methylation (2′-O-Me), and 10 methylated bases [88]. Modifications are primarily found in the functional regions of the ribosome and are induced by snoRNPs wherein snoRNAs complementary to specific rRNA sequences determine the methylation site [89, 90]. snoRNAs can be divided into C/D or H/ACA box-containing snoRNAs [91]. C/D box snoRNAs predominantly undergo 2′-O-Me modification, while H/ACA box snoRNAs undergo substitution with *Ψ* [92]. rRNA modification alters the secondary and tertiary structure of ribosomes that is important for ribosome biogenesis and function [93]. Differential modification of particular rRNA sites results in ribosome heterogeneity.

Fragile X mental retardation protein (FMRP) is an RNA-binding protein that is important for neuronal development and differentiation. In animal and human stem cells, FMRP maintains pluripotency, regulates cell fate, and determines the speed of generating neuronal lineage-committed cells [94–97]. FMRP has been shown to function predominantly in the cytoplasm; however, recent evidence has demonstrated its role in the nucleus [98, 99]. In the nuclei of human embryonic stem cells, FMRP directly interacts with C/D box snoRNAs and results in the 2′-O-Me modification of rRNA, thereby causing ribosome heterogeneity by affecting rRNA folding and ribosomal assembly [100, 101]. In the cytoplasm, FMRP identifies 2′-O-Me-modified ribosomes to enable specific translation of its target mRNAs [101, 102]. FMRP promotes the expression of genes involved in stem cell intracellular pathways, such as mTOR, PI3K, ERK, and Gsk3*β* [103–106].

Fibrillarin is a protein that is involved in proliferation [107], cancer [108], and stem cell differentiation [43]. Fibrillarin is enriched in the DFC region of the nucleolus and contains an N-terminal domain rich in glycine and arginine residues (namely the GAR domain), a central RNA-binding domain comprising an RNP-2-like consensus sequence, and a highly conserved C-terminal helical domain that may act as methyltransferases [109, 110]. The human GAR domain enables fibrillarin-interacting pre-RNAs to process nascent 47S pre-rRNAs and demarcate the DFC region. As a part of C/D box snoRNPs, FBL catalyzes the 2′-O-Me of rRNAs to regulate ribosome biogenesis and translation [111, 112]. Thus, fibrillarin functions in pre-rRNA processing and modification, thereby regulating ribosomal biogenesis. It can also enhance the activity of RNA polymerase I. Nop1, the yeast homolog of fibrillarin, also processes pre-RNAs, especially 18S rRNA. Fibrillarin has been reported to be overexpressed in mouse embryonic stem cells and maintains pluripotency state even in the absence of LIF [43]. During stem cell differentiation and neurogenesis, fibrillarin is downregulated and may affect the 2′-O-Me modification of rRNAs to regulate ribosome biogenesis with modified translational specificity such that IRES-containing mRNAs (e.g., *cMYC*, *FGF1*, and *VEGFA*) are preferentially translated instead of 5′-capped transcripts [43, 107, 108].

### 1.7. RP Heterogeneity in Stem Cells

Differences in RP composition and isoform lead to ribosome heterogeneity [113] that enables the recognition of sequence-specific elements or structures in mRNAs and selective expression [2, 114, 115]. Various RPs express to different extents in different tissues of the developing mouse embryo [116].

Quantitative mass spectrometry was used to measure the RP abundance and identify heterogeneous compositions of translationally active ribosomes in mESCs [2]. Ribosomes containing RPS25 or RPL10A translate specific transcript subpools, including mRNAs encoding key components in metabolism, the cell cycle process, and development, while the depletion of RPL10A does not affect the overall polysome profiles but reduces translation efficiencies of mRNAs associated with metabolism [2]. The heterogeneous RPs identified by SRM are located on the surface of the ribosome in important functional regions including the mRNA exit tunnel and the L1 stalk and thus directly interacts with mRNAs [117, 118]. RPL10A directly interacts with the IRES and engages the 80S ribosome independent of some or all initiation factors to achieve translational regulation of mRNAs, highlighting the importance of cis-regulatory elements in selective mRNA translation [2, 119, 120].

Diamond-Blackfan anemia is a special hematological disease. Patients present with a decrease in the population of erythroid precursors and progenitors in the bone marrow that is caused by heterozygous loss-of-function mutations in one of 18 different RP genes (e.g., RPL11, RPS19), thereby resulting in RP haploinsufficiency [121, 122]. Knockdown of RPL11 or RPS19 reduces IRES-mediated translation, especially of Bag1 that protects GATA1 from caspase-3-mediated cleavage during terminal erythroid differentiation [123–125]. RP mutations reduce the key lineage-determining hematopoietic transcription factor GATA1 mRNA in Diamond-Blackfan anemia [125].

Mutations in RPL21 are linked to stem cell-specific defects, such as loss of body hair [126]. RPL38 mutant embryos show no change in global protein synthesis but selectively affect the translation of a subset of Homeobox mRNAs [116].

Collectively, these findings suggest that RPs are regulated to confer a new layer of specificity in the control of gene expression, mammalian development, and stem cell biology.

### 1.8. AFs Interact with RPs to Regulate Stem Cell Function

RP synthesis is closely linked to other biological processes [127]. RPs are translated in the cytoplasm by preexisting ribosomes following which they enter the nucleoli and bind to rRNA to form ribosomes. RPs may play selective roles in eukaryotic ribosomes during cellular homeostasis and development [114]. Some AFs directly interact with and stabilize RPs, while others associate with DNA to stimulate transcription. UBA52 encodes a fusion protein of ubiquitin and RPL40 that is important for embryonic development. The RPL40 cleaved from UBA52 is important in protein biogenesis and forms a ribosomal complex with ubiquitin cleaved from UBA52. Efficient protein synthesis requires the cleavage of RPL40 from the fusion protein [128].

Bmi1 is a member of the polycomb group of proteins that bind to the promoter of target genes and induce epigenetic modifications in the chromatin to regulate cancer and stem cell biology [129–131]. Bmi1 affects the proliferation and differentiation of HSCs as well as other stem cells, such as mesenchymal stem cells and neural stem cells [132, 133]. In K562 cells, Bmi1 binds to the promoter of ribosomal genes, such as *RPL5*, *RPL1*, *RPL23*, *RPS14*, and *RPS19*; thus, a loss in this interaction downregulates ribosomal proteins and results in impaired ribosome biogenesis, thereby reducing global translation efficiency [134, 135]. Bmi1 promotes the transcription of RPs by recruiting active histone marks including H3K9ac and H3K4me3.

Runx1, another transcription factor, binds to the promoters of RP-encoding genes and rDNA repeats to regulate the transcription of rDNA and ribosomal biogenesis in HSPCs [65, 136]. Runx1 forms the core ribosomal promoter element with RUNX1, GATA2, and FLI1 that affects ribosomal biogenesis in conjunction with cooperative hematopoietic transcription factors [137, 138]. Runx1 is regulated by the global regulator of ribosome biogenesis, Myc [139]. Moreover, Bmi1 directly interacts with RUNX1 to recruit polycomb repressor complex 1 to regulate ribosome biogenesis and assembly [140].

**Figure 2:**
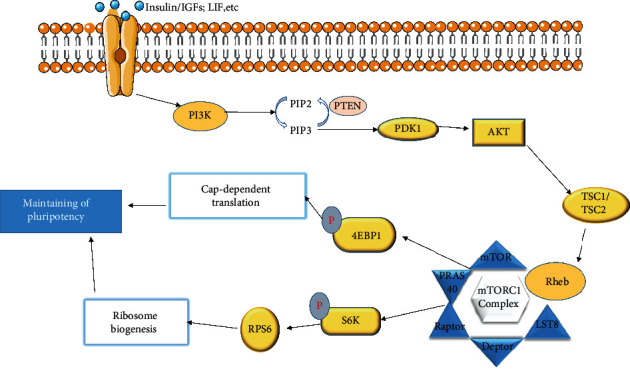
PI3K/AKT/mTORC1 signaling involved in translation and pluripotency. PI3K is activated by cytokines, such as LIF, Wnt, and growth factor receptors, to induce cell proliferation and regulate mTOR signaling to maintain pluripotency. This primarily involves the mTORC1 complex that phosphorylates S6K and 4EB-P1. Phosphorylated 4EB-P1 relieves suppressed translation by stimulating cap-dependent translation; phosphorylated S6K increases the levels of ribosomal protein S6 that is important in the biogenesis of 40S subunits. This helps maintain stem cell pluripotency by enhancing ribosome biogenesis to promote the translation of specific stem cell genes.

The phosphatidylinositol-3-kinase (PI3K)/Akt and mammalian target of rapamycin (mTOR) signaling pathways (PI3K/Akt/mTOR) are pivotal for cell growth and survival [141–144]. Cells can be reprogrammed by activating IGF1/AKT/mTOR signaling and increasing the translation of RPs in cells depleted of MeCP2 (Figure 2) [145]. mTOR phosphorylates downstream effectors, including S6 kinase (s6K) [146] and eukaryotic initiation factor 4E (eIF4E) binding protein 1 (4E-BP1) [147, 148]. eIF4E inhibits translation, while mTOR-phosphorylated eIF4E relieves translational suppression to promote cap-dependent translation [148]. The phosphorylation of S6K promotes the biogenesis of RpS6 (component of the 40S subunit) and translation [149, 150]. mTOR is important for the development of ES cells and can be regulated by PI3K signaling involved in ES cell pluripotency [151–153].

### 1.9. Ribosome Assembly and Transport in Stem Cells

PDCD2 is a conserved protein in eukaryotes that is present in mouse ES cells and other rapidly proliferating cells, such as cancer cells, and detected in abundance (if at all) in differentiated or slow-growing cells [154–157]. Zfrp8, the homolog of PDCD2 in *Drosophila*, functions in the maintenance of HSCs [158]. PDCD2 is a member of TYPP domain-containing proteins (TSR4, YwqG, PDCD2L, and PDCD2), among which TSR4 regulates rRNA processing and ribosome maturation [159]. Zfrp8/PDCD2 directly interacts with the 40S ribosomal subunit via RpS2, thereby regulating the cytoplasmic levels of RpS2 and stability of the 40S subunit [160]. The 40S subunit consists of more than 30 RPs that bind to numerous non-RPs to regulate translation, subunit assembly, and nucleocytoplasmic transport [161, 162]. Thus, Zfrp8/PDCD2 plays a key role in translation; however, it is not essential during general translation [160]. Zfrp8/PDCD2 can recruit different RNA-binding proteins, such as FMRP/Fmr1 and NUFIP1/Nufip (nuclear FMRP-interacting protein), form mRNA-RNP complexes that bind specifically to the 40S subunit, and spatiotemporally regulate target gene expression [163–165]. Zfrp8/PDCD2 also regulates the translation of protein-coding genes by promoting nuclear export of the mRNAs [160]. Thus, Zfrp8/PDCD2 is important in ribosome assembly and regulates the transport of specific mRNAs to maintain properly functioning stem cells.

## 2. Conclusions

Ribosomes are tools that are important for translation in different kinds of cells. However, recent research has shown that it exists in heterogeneous forms to differentially regulate gene expression [2]. Ribosome biogenesis is a very complex process. Although the basic steps of ribosome synthesis are conserved [166], there are various factors that can regulate the different processes [167] to modulate the translation efficiency of specific genes. Most of these factors are highly expressed in stem cells; knockout or mutation affects stem cell function and leads to cell death. Ribosome heterogeneity is when ribosomes have different composition, such as rRNAs, RPs, and AFs, and allows the selective translation of mRNAs to generate the appropriate types and amounts of proteins needed to regulate cellular function to the environment. Specific features in mRNAs, such as cis-elements, are recognized by specialized ribosomes, thereby enabling selective translation [2]. What is more, mRNA recognition and translation by the ribosome are based on combinatorial sets of RNA–RP interactions; thus, ribosome heterogeneity and its role in translational control may be mainly determined by RP composition and modification [115]. We know that there is a long way to go to decipher the heterogeneity ribosome, and recently, research discovered that stem cells and differentiated cells express different subsets of tRNAs [168, 169].

Stem cells differentiate into lineage-committed cells that proliferate to form specific tissues, organs, and systems in our body, thereby highlighting their importance as ideal sources for repair of damaged cells and tissues. Owing to the limited abundance of stem cells, Yamanaka and colleagues expressed four specific genes (*OCT4*, *KLF4*, *SOX2*, and *CMYC*; OSKM) to reprogram differentiated cells into induced pluripotent stem cells [170]. However, since reprogramming is an inefficient process, there is ongoing research on the identification of factors that accelerate reprogramming [171, 172]. Numerous studies have shown that the presence of long noncoding RNAs (lncRNAs) promote the maintenance of stem cell function [173]. LncRNAs, such as Peblr20 and SNHG14, significantly improve reprogramming efficiency [174, 175]. This review focuses on the diversity of ribosomes associated with the translational control of stem gene expression and identification of specific recognition elements in the mRNAs associated with stemness. AFs in stem cells improve the efficiency of ribosome biogenesis and promote the translation of stem cell-related genes. Thus, using these AFs with the factors involved in reprogramming (lncRNAs, proteins, etc.) will promote ribosome synthesis and improve reprogramming efficiency. However, further research is required on the mechanisms by which ribosomes specifically regulate the expression of selective stem cell-related genes. Addressing this will pave way for a new direction in stem cell research that will help stimulate stem cell reprogramming and promote the clinical application of stem cells.

## Data Availability

No data were used to support this study.
